# P22 Will the real scleroderma please stand up?: a case of a rare scleroderma mimic in ED

**DOI:** 10.1093/rap/rkad070.043

**Published:** 2023-09-27

**Authors:** Sasha Saadia Ali, Sook Yan Lee, Arti Mahto

**Affiliations:** King's College Hospital, London, United Kingdom; King's College Hospital, London, United Kingdom; King's College Hospital, London, United Kingdom

## Abstract

**Introduction:**

Scleroderma is a rare autoimmune connective tissue disorder that is largely considered the disease prototype for sclerosing skin conditions. However there are other rare conditions that can mimic scleroderma (1). Differentiating between these can be challenging, especially in the early stages of the disease. However, ascertaining the correct diagnosis is crucial as timely intervention and treatment are important in both scleroderma and the so-called ‘scleroderma mimics’ (2). Additionally, incorrect treatments can expose patients to immunosuppression and its inherent complications. Here we present the case of a scleroderma mimic, scleromyxoedema and discuss the implications of the diagnosis and limited management options.

**Case description:**

A 56-year-old man presents with a 10-day history of arthralgia and pruritic rash. The rash began on his face, and spread to involve his posterior neck, upper back, anterior chest and upper limbs, with a hardening of the skin over his back and fingers. He reported symmetrical joint pain affecting his PIPS, MCPs and wrists. He had no past medical history of note. He was born in Colombia, and moved to the UK in the last five years having spent time in Spain in his early 20’s. There was no other recent foreign travel. On examination, has had grossly swollen digits with mild oedema in the upper limbs. He had solid skin-coloured papular lesions and dermal nodules overlying erythematous thickened oedematous skin. Synovitis was noted in his MCPs and wrists. There was no nailfold capillary change or symptoms of Raynaud’s.

Routine bloods were unremarkable with normal inflammatory markers, ANA, ENA, ANCA, RF and complement. There were no monoclonal bands on serum electrophoresis and serum light chains were normal. CT chest abdomen and pelvis showed no malignancy. Histopathology of his skin showed extensive mucin deposition throughout the dermis with separation of the collagen fibres and scattered stellate fibroblasts, suggestive of scleromyoxoedema. Other localised forms of lichen myxoedema were deemed unlikely as his thyroid function and thyroid US scan were both normal.

He was started on 40mg prednisolone, which was gradually weaned over 8 months. This improved his skin, though the persisting thickening of his eyelid had to be managed with twice daily pimecrolimus 1% cream. He was also commenced on topical steroids, fexofenadine and amitriptyline for pain. As this condition is strongly associated with monoclonal gammopathy, he was referred to the haematology team for further investigation, though he declined a bone marrow biopsy to investigate for plasma cell clones.

**Discussion:**

Scleromyxoedema is a rare (incidence and prevalence unknown) condition which usually occurs in middle aged adults. It is characterised by a diffuse papular cutaneous skin eruption which is strongly associated with a monoclonal gammopathy and may have systemic features of dysphagia, oesophageal dysmotility, myopathy and even cardiopulmonary and CNS involvement. The pathogenesis remains unknown however circulating cytokines which stimulate glycosaminoglycans and fibroblasts may play a role.

Our patient presented with skin thickening and joint pains, however the papular skin eruption in the absence of Raynaud’s and nailfold changes prompted urgent dermatology review and skin biopsy eventually confirmed the diagnosis. The clinical course of scleromyxoedma is usually chronic and progressive, with variable success of therapies including IVIG, cyclophosphamide, methotrexate and thalidomide. Reassuringly this patient’s skin condition and synovitis seemed to clear with high doses of prednisolone without the need for further immune supressing agents after 8 months, and no recurrence was noted at follow up 12 months later. As his skin has not fully cleared with mild eye lid involvement, he remains under the careful watch of the dermatology and myeloid specialist teams.

This case is interesting as it represents a rare mimic of scleroderma which was promptly recognised and treated by the appropriate specialists , which may have improved this patient’s long term outcome. Whilst scleromyxoedma is usually associated with a monoclonal gammopathy and can be highly supportive of its diagnosis, the presence of the typical sclerodermoid eruption with histopathology confirming mucin deposition and the absence of any thyroid disorder has confirmed the diagnosis in this case (3). The recurrence of scleromyxedema is common after withdrawal of therapy and long-term treatment with IVIG and other immunosuppressives may be required. Reassuringly, he remains under follow up with haematology who are monitoring him for the development of a monoclonal band.

**Key learning points:**

The case highlights the importance of recognising a scleroderma mimic early in the disease course as it allows prompt triage to the most appropriate specialist teams and early treatment which may have improved the patient’s outcome.

There remains a paucity of data on treatment options for this condition, and treatment decisions are currently primarily guided by case reports and case series. This patient responded well to steroids but did require high doses over a prolonged period. IVIG would have been a good option here, but would involve individual funding request applications which has become more difficult to secure in the U.K. This case highlights the need for further research into this condition, which would add to the growing literature base to help guide the management of these patients.

A patient centred approach, with patient involvement in the decision-making process as seen in this case, is key to managing any disease but is especially vital in rare conditions where there are no consensus guidelines on treatment. If riskier strategies are needed in the future, the multidisciplinary team along with this patient would have to balance the potential risks associated with these treatments.

